# A Modern Historical Perspective of Schroth Scoliosis Rehabilitation and Corrective Bracing Techniques for Idiopathic Scoliosis

**DOI:** 10.2174/1874325001711011452

**Published:** 2017-12-29

**Authors:** Kathryn Moramarco, Maksym Borysov

**Affiliations:** 1 Scoliosis 3DC, 3 Baldwin Green Common #204, Woburn, MA 01801, USA; 2 Maksym Borysov, PT, CPO, Orttech-Plus Rehabilitation Services, Kharkov, Ukraine

**Keywords:** Scoliosis, History, Schroth, Rehabilitation, Bracing, Idiopathic

## Abstract

The treatment of scoliosis has a long history dating back to Hippocrates and his luxation table. In recent history, conservative rehabilitation treatment methods have come and gone. Some have had more longevity than others and currently there are only a handful of these “schools” for rehabilitation in existence.

What is important to note in this twenty-first century world is that any approach to bracing or scoliosis rehabilitation must strive for a correction effect and be as user-friendly as possible. Patients look to achieve some measure of success, whether it be halted Cobb angle, improved breathing function, decreased rotation, or postural improvement *via* trunk symmetry.

Katharina Schroth created her method in 1921 as a result of self-analysis of her own imperfect scoliotic torso and the effect on it as she altered her breathing patterns. It was from these observations and self-experimentation that she devised her rotational angular breathing method. Subsequently, the Schroth method evolved under the leadership of her daughter, Christa Lehnert-Schroth P.T., and grandson, Dr. Hans-Rudolf Weiss. Collaboration with Dr. Jacques Chêneau led to a new Schroth method compatible scoliosis bracing approach. The most recent advancement of Chêneau bracing is the Gensingen Brace® (GBW). Gensingen braces have an asymmetric design and rely on Schroth principles of correction in a smaller, lighter, more wearer-friendly brace. Each brace is designed to be a complementary supportive orthosis. It may be used independently, or in conjunction with Schroth exercise protocols.

## 1 INTRODUCTION

**Fig. (1):**
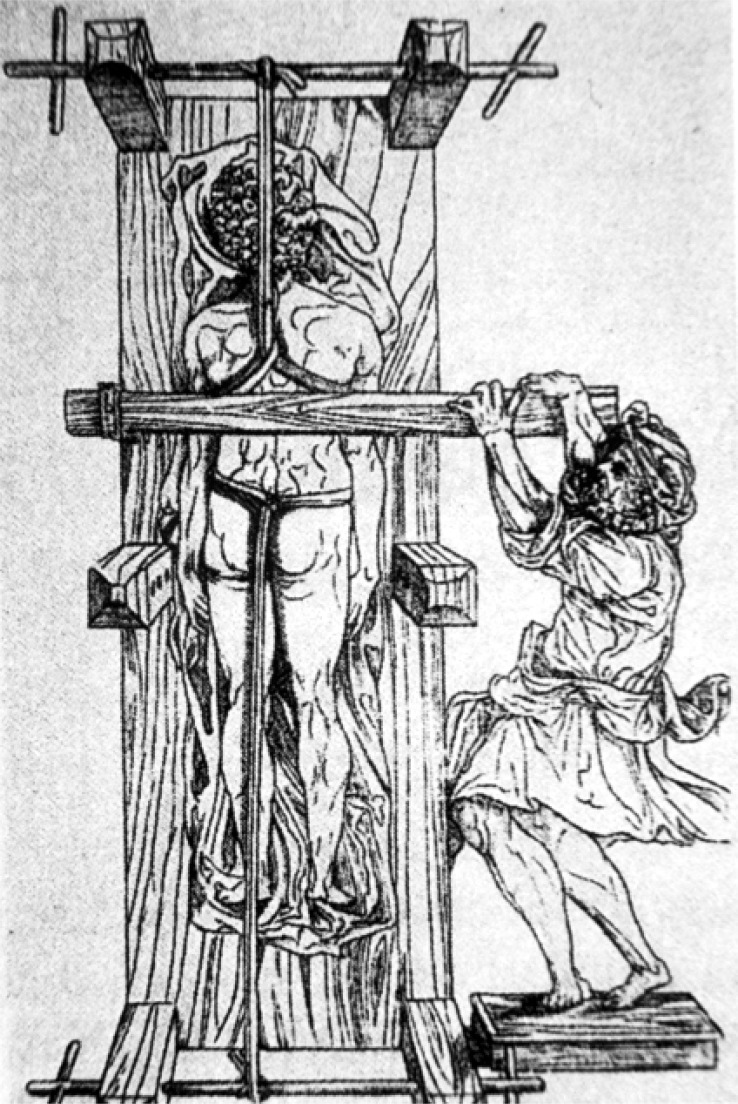
Hippocrates Luxation Table [5].

**Fig. (2):**
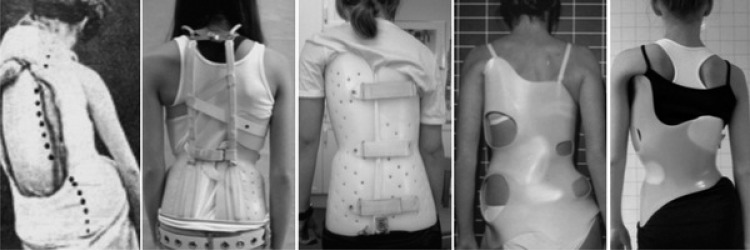
Scoliosis brace examples: past to present: From left, Abbott 1910, Milwaukee Brace, Boston Brace, Chêneau Brace -1990s, Gensingen Brace (Chêneau) - 2016. [Courtesy of Dr. HR Weiss and Scoliosis 3DC, USA].

The treatment of scoliosis has a long history dating back to Hippocrates and his luxation table (Fig. **1**) [1]. In recent times, bracing has been the predominant mode of attempted correction [2-7] for the conservative treatment of idiopathic scoliosis (Fig. **2**). The industry standard for a successful bracing outcome is considered by most practitioners to be curve stabilization [7]. Unfortunately, not all patients are able to achieve this benchmark when wearing a thoracolumbosacral orthosis (TLSO) since a subset of patients continue to progress despite bracing. When scoliosis is diagnosed or progresses to a 40º - 50º Cobb angle or more, the solution typically offered to prevent further progression is a complex surgical intervention usually consisting of multi-segmental fusion [8]. Despite its limitations, bracing has been the best hope for the thousands of patients diagnosed annually with spinal curvatures. Several European countries also have “schools” of exercise specific to treating scoliosis [9]. These methods exist in an effort to help patients manage the condition on their own. For adolescents, scoliosis rehabilitation is often an adjunct to bracing, or for some, an alternative to bracing. In recent history, several methods have come and gone with some having more longevity than others. Currently, there are only a handful of these exercise-based schools.

## 2 HISTORICAL PERSPECTIVE OF THE SCHROTH METHOD

Many patients today are beginning to reject the long-standing watchful waiting approach and prefer to seek knowledge about effective curve management *via* exercise rehabilitation. Through exercise rehabilitation, the patient should gain an understanding of their unique spine and the postural modifications needed to attempt improved symmetry and spinal stabilization. Since, idiopathic scoliosis typically develops during early adolescence, young patients learn curve management techniques that can be used over the course of their lifetime, if needed [5]. Concepts should be easy to incorporate and absent of inciting pain–even when the goal is to induce correction.

**Fig. (3):**
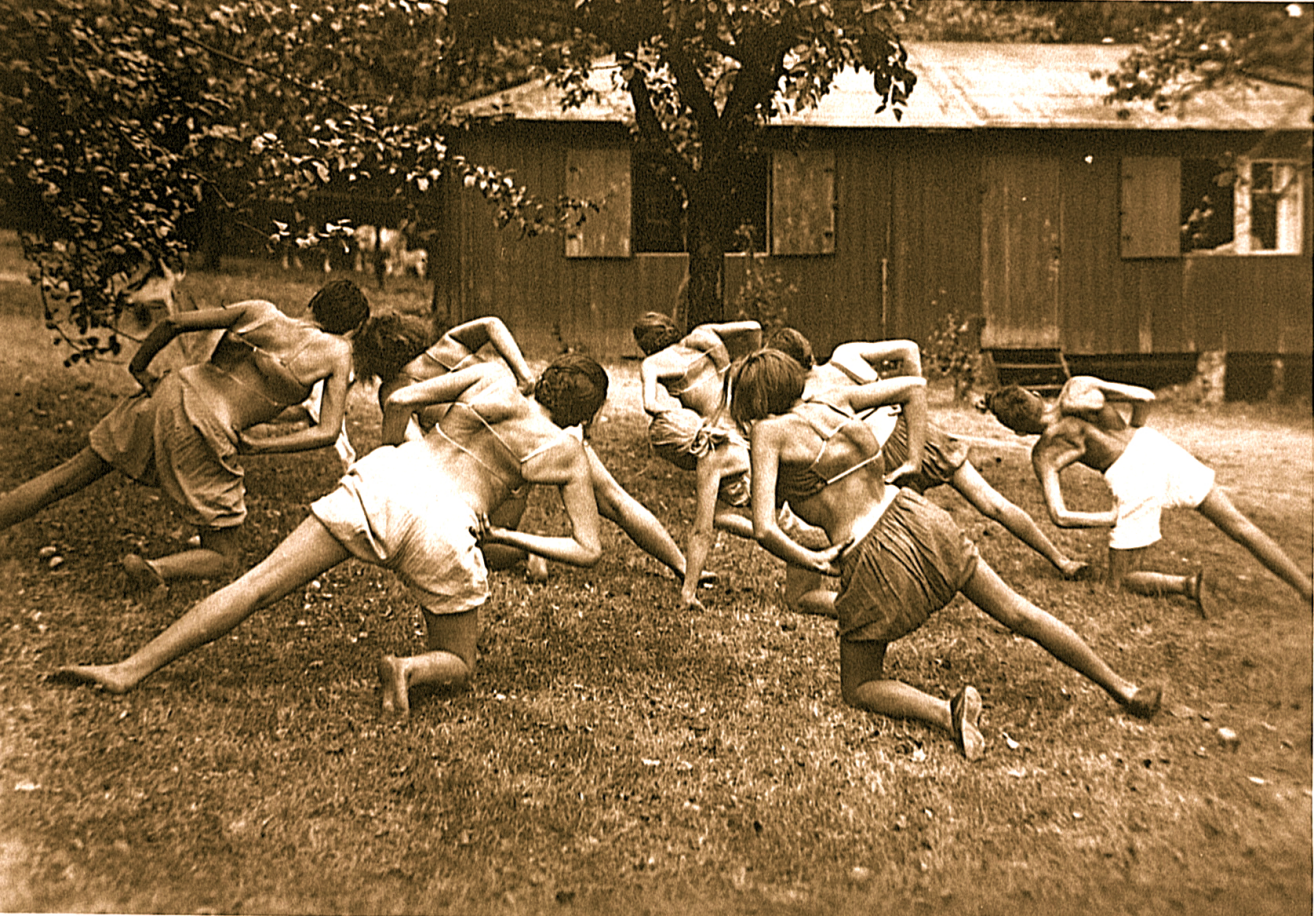
Patients performing Schroth exercise at the Meissen Institute of Katharina Schroth, circa 1930s. [From the photo archives of Christa Lehnert-Schroth].

Today, the most widespread method of physical rehabilitation for the treatment of scoliosis and hyperkyphosis is the Schroth method of Germany. Schroth is a corrective, evidence-based approach in use since 1921 (Fig. **3**). The method and its developments are the culmination of the professional endeavors of three generations of one family. Its success is credited to its proprietary Schroth rotational angular breathing (RAB) technique [10]. With the Schroth method, postural corrections are learned and applied. Patients alter and correct the scoliotic breathing pattern and work to improve individual postural perceptions [5, 10-14]. These elements can play a pivotal role in scoliosis management. Compliant patients may achieve positive outcomes for scoliosis in terms of halted or reduced Cobb angle measurements, decreased angle of trunk rotation, improvements in breathing function, and/or a more balanced posture [5].

**Fig. (4):**
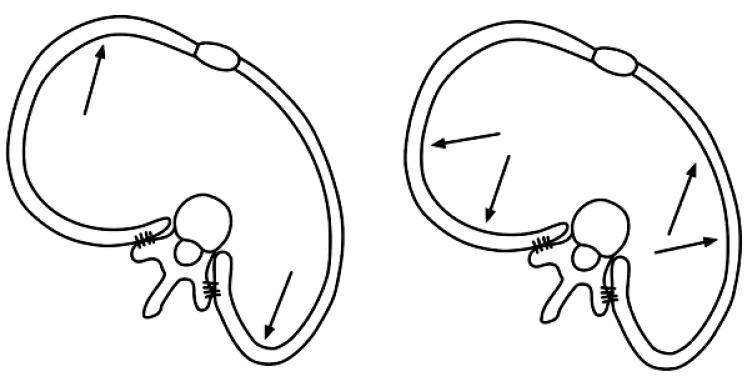
Example of the altered breathing mechanics with scoliosis (L); example of Schroth rotational angular breathing to improve breathing mechanics (R) [adapted from 10].

The developer of the original Schroth method, Katharina Schroth, created the method after analyzing her own imperfect scoliotic torso and altering her breathing patterns to observe the effect [10]. As a result of this self-experimentation, she developed her rotational angular breathing method (Fig. **4**). By observing the effect in a mirror, Katharina would go on to practice and perfect the corrective movements and the derotational breathing technique that she devised.

This “mirror monitoring” took on an important role in the Schroth method and it is still used in the Schroth Best Practice® protocols of today – the version of the Schroth method endorsed by third generation Schroth family member Dr. Hans-Rudolf Weiss (5). Mirror monitoring allows the patient to synchronize the corrective movements and postural perceptions and receive immediate visual feedback [5, 10-14]. The breathing and functional corrections incorporated are evidenced in Katharina Schroth’s early writings [10-14].

**Fig. (5):**
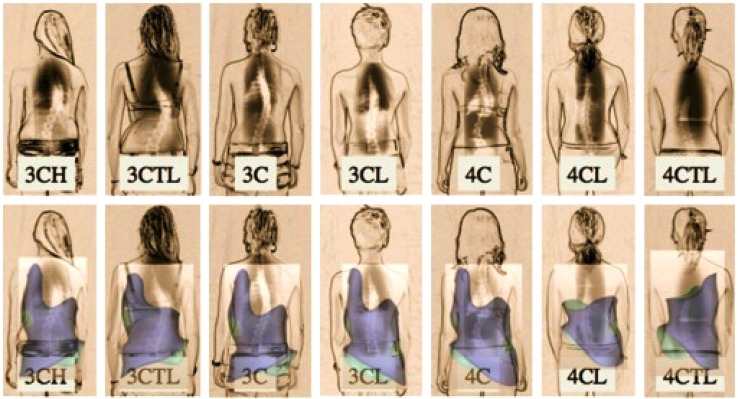
The Augmented Lehnert-Schroth Classifications. [Courtesy of Dr. HR Weiss].

In the decades that followed, Christa Schroth (later Christa Lehnert-Schroth) played an important role in advancing the method her mother created. By the 1970s, she had recognized the importance of the lumbosacral (counter-) curve (4^th^ curve) for pattern-specific postural correction [14]. She went on to introduce a simple classification system for the treatment of scoliosis that is still in use today (Fig. **5**) [14]. Her classification system contributed significantly to the advancement of the method. It is described in her book, *Three-Dimensional Treatment for Scoliosis*, which was initially published in 1973 (in German) and later translated to several other languages, including English [10]. Lehnert-Schroth continued to be active in advocating for the method until her death in 2015 [5]. Just prior to this, she co-authored an updated book titled, “Schroth Therapy: Advancements in Conservative Scoliosis Treatment” with her son Dr. Hans-Rudolf Weiss, and colleague Marc Moramarco, D.C [5].

Aside from Schroth, there are also other European “schools” for scoliosis that attempt to induce a corrective effect *via* exercise rehabilitation. Of them, only the Lyonnaise school of France has had a longer history than the Schroth method [9]. The Lyon method is still in use today in France. SEAS is another approach from Italy, which was originally derived from the Lyonnaise school and has evolved over time to include treatment principles from other methods as well [9, 15]. In Poland, Dobosiewicz’s method for the treatment of scoliosis (Dobomed) has been in existence since 1979. With these exercises, a global kyphosis is induced. The breathing exercises incorporated have been derived from the Schroth program [9] as they are similar to Schroth rotational angular breathing. Another approach, developed by Dr. Min Mehta in 1984, is side-shift. The technique is used today by therapists at the Royal National Orthopaedic Hospital [9]. FITS: Functional Individual Therapy of Scoliosis, is a technique from Poland. FITS also focuses on asymmetric correction and is reported to be a blend of several techniques, which, in practice, the developers found to be beneficial for patients [9]. Others have proposed yoga and scolio-pilates as modes of rehabilitation for scoliosis, but these claims are currently without evidence. While yoga is an excellent exercise approach for the symmetric torso, for an individual with an asymmetric body configuration there are movements that are contraindicated [5].

Over time, Schroth ‘spin-offs’ have emerged. These “Schroth schools” are primarily from therapists and practitioners trained by Christa Lehnert-Schroth or at the Asklepios Katharina Schroth Clinic under the tutelage of Dr. Weiss. One branch is the Barcelona school. Today, this school has implemented new nomenclatures [9] and appears to have distanced itself somewhat from the original principles [10]. This version, and others, differ from the current Schroth Best Practice® concepts on a number of fronts [5].

**Fig. (6):**
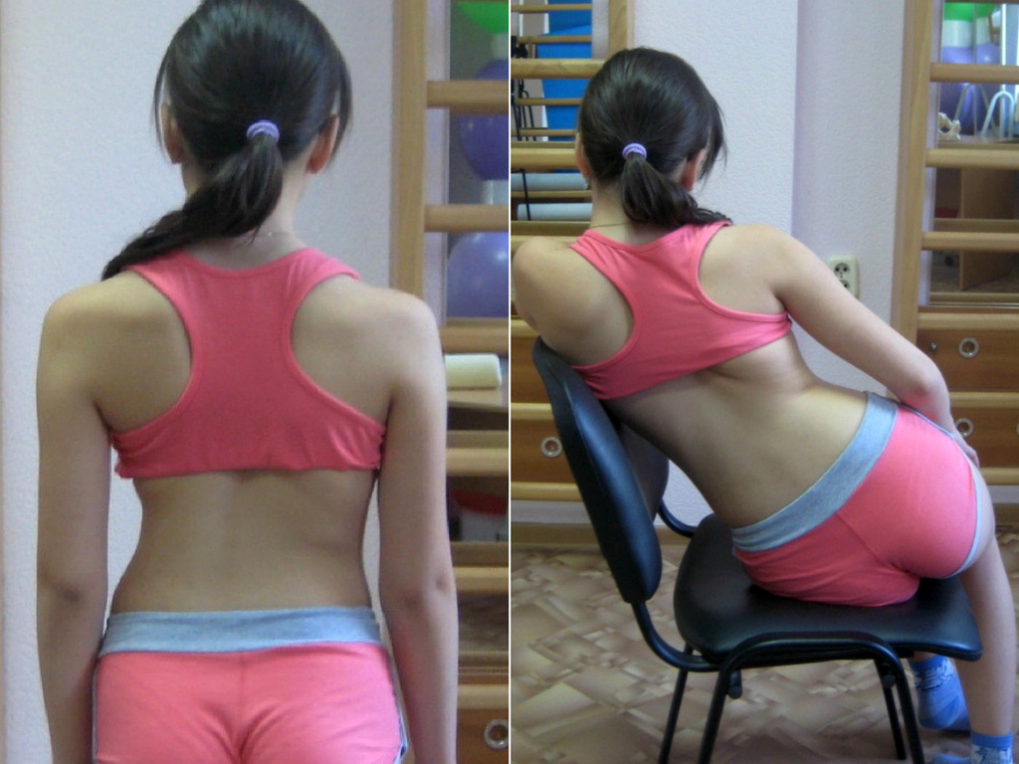
Example of a curve-pattern-specific activity of daily living [Courtesy of Orttech Plus, Ukraine].

Schroth Best Practice® retains the original Schroth concepts but introduces new forms of postural education [5, 14]. To differentiate from other schools of Schroth currently in use, Schroth Best Practice® uses the term pattern-specific scoliosis rehabilitation (PSSR). In recent years, under Dr. Weiss’s direction, the newest Schroth concepts have spread beyond the German border to patients around the globe. While the original Schroth exercises have been proven to be effective [16-18], the Schroth Best Practice® updates take those original concepts but make rehabilitation easier for the patient to understand and integrate into daily life. Correction of the sagittal plane *via* physiologic® exercises and active self-correction during curve-pattern specific activities of daily living (ADL) are important components (Fig. **6**). These simple yet effective add-ons are based on the most current scoliosis research [5, 19-21]. With these updates, patients participate in a program of efficient, experiential learning over the course of a few days, which enables complete independence from the therapist [5] by the conclusion of instruction.

In the 1990s, the Schroth family sold their interest in the 150-bed in-patient Katharina Schroth clinic to the Asklepios hospital group but retained control of medical administration. It was during his tenure as medical director of the clinic that Dr. Weiss recognized the necessity for the continuing evolution of the Schroth method. In 2008, he left the clinic to practice independently and develop improved protocols for rehabilitation and bracing.

Both the original and Schroth Best Practice® protocols have a long record of research and evidence [16-26]. Consequently, Dr. Weiss has helped facilitate the expansion of the technique beyond Germany. Since then, other important studies have emerged from Turkey [20, 26] as well as recent randomized-controlled studies on Schroth [17, 18]. Today, Weiss works with a board of affiliates, each dedicated to teaching the Schroth method [5] protocols and its updates to patients and practitioners internationally.

## 3 MODERN HISTORICAL PERSPECTIVE OF BRACING

**Fig. (7):**
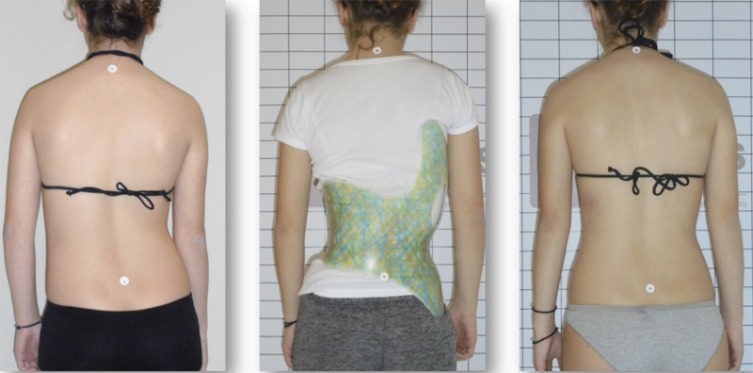
The Gensingen Brace® is designed with the goal of improving postural symmetry. [Courtesy of Dr. HR Weiss].

To date, bracing has been the most important non-surgical treatment modality used for the treatment of scoliosis [7]. Some of the aforementioned exercise methods have corresponding TLSOs [9]. Chêneau-style braces, from Europe, are linked with the Schroth method and are rapidly gaining popularity. They differ from their American counterparts, which according to Fayssoux *et al.*, “are to prevent progression of deformity and to obviate the need for spinal fusion, not to improve the deformity” [7]. In contrast, Chêneau braces aim to improve postural symmetry (Fig. **7**) and help the patient achieve some degree of spinal correction by the time brace weaning occurs [5]. Furthermore, Chêneau braces, with their anterior closures, are easier for patient to manage independently. As a result, Chêneau concept braces are spreading globally.

Still, the most commonly used brace for scoliosis is the Boston (concept) brace from the United States [27-30]. Boston braces are rigid, symmetric braces with interior pads and posterior closures. The brace was developed in the city for which it is named and (first) described by Watts *et al.* in 1977 [27]. Other braces used in the U.S. are also named for the locales where they originated and include the Milwaukee brace and the Wilmington brace [7]. For nighttime wear, the Providence brace and Charleston brace are often recommended [7, 31, 32].

The BrAIST study, an RCT published in 2013, helped to validate the efficacy of rigid bracing. The study reported that the Boston brace was successful in preventing surgery for 72% of compliant patients and that the rate of treatment success was positively associated with hours of daily brace wear [33]. One strength of this study was the use of an implanted sensor to monitor patients’ brace wear-time. In terms of correction effect, moderate in-brace corrections have been reported with the Boston brace [28, 29]. Another multi-center prospective controlled study by Nachemson and Peterson also demonstrated that 70% of the treated cohort was non-progressive [30].

The Milwaukee brace is another U.S. brace. Blount and colleagues developed this brace in 1958 with contributions by Dr. John Moe [34, 35]. In general, the in-brace corrections achieved with this brace have been unremarkable, with the exception of a study by Maruyama [36]. Today, the Milwaukee brace is used in some countries internationally, but less so in North America – where it is mainly used for high upper thoracic curves [7].

In France, Pierre Stagnara created the Lyon brace in 1947 [9]. It was the first 3D adjustable correction brace created from a plaster cast. The Lyon brace attempts elongation of the patient’s torso with equal distribution of forces on the right and left to attempt global detorsion of the spine. Elongation requires precise adjustment of the brace while a child is growing [37]. The Lyon brace has undergone several developments in recent decades. The newest is the ‘Lyon ARTbrace’ [9]; a device produced and fit primarily in France.

Another 3D brace from Europe, the Chêneau brace, was first studied in 1979 [38]. The brace developer, Dr. Jacques Chêneau of France, derived his concept from the Abbott technique of casting. The first long-term results were published in 1985 [39]. In 2005, a prospective controlled study was published on a homogenous group of patients at high risk for progression. The results showed that 80% of the braced population was non-progressive [40]. To better understand the Chêneau bracing concepts, it is helpful to understand how Chêneau bracing is intertwined with the Schroth method. While there was never a specific “Schroth Brace” in the early history of the technique, the development of Chêneau bracing was influenced significantly by the three-dimensional German treatment [5].

Dr. Chêneau first visited Katharina Schroth and Christa Lehnert-Schroth in Germany in the 1970s and quickly recognized the potential that Schroth principles could have on scoliosis bracing. As a result, he made subsequent visits [5] to understand more about the Schroth breathing pattern principles from the mother-daughter team. Chêneau also became familiar with and embraced the Lehnert-Schroth pattern classifications [41]. He began to apply these classifications in his brace designs by creating voids, or openings, at strategic places so that patients could benefit from Schroth corrective breathing in-brace [38].

**Fig. (8):**
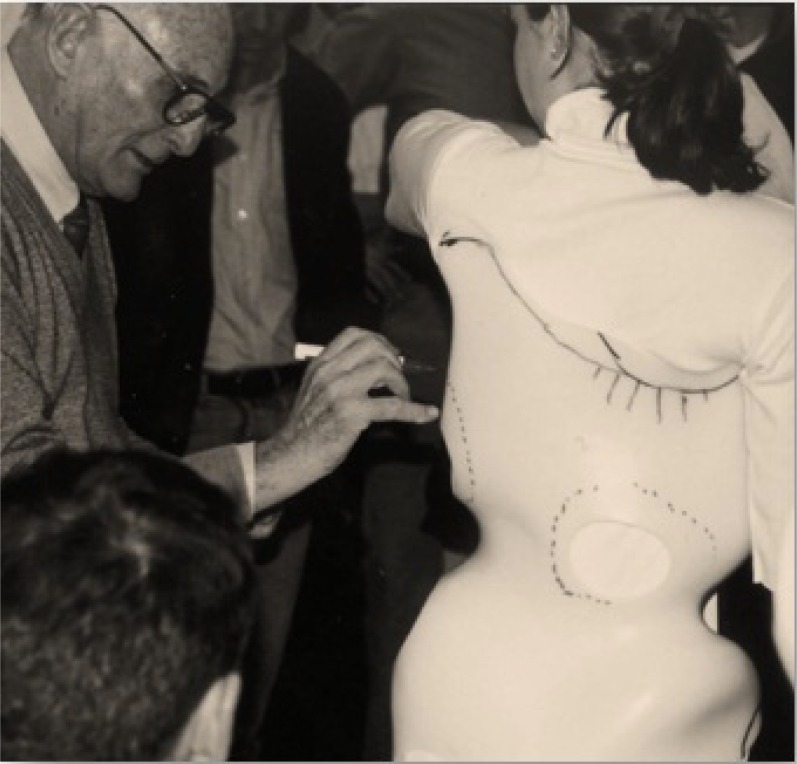
Dr. Chêneau conducting a workshop at the Katharina Schroth Clinic, Bad Sobernheim, Germany, circa 1990s. [From the photo archives of Christa Lehnert-Schroth].

In the 1990s, Weiss advanced the Schroth-Chêneau relationship by initiating annual workshops attended by physiotherapists and orthopedic technicians (Fig. **8**), including Dr. Rigo of Spain [5]. At these workshops, Dr. Chêneau demonstrated his technique of producing Chêneau orthoses from a plaster cast [38] and discussed his on-going developmental work.

**Fig. (9):**
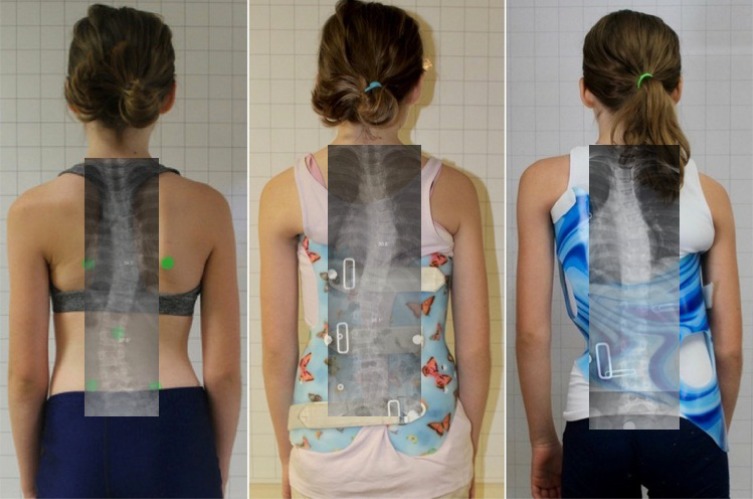
Comparison of an 11-year-old patient wearing a Boston Brace (center) and a Gensingen Brace® (right) made six weeks apart (2016). The Gensingen Brace® strives for improved in-brace corrections for improved outcomes. [Courtesy of Scoliosis 3DC, USA].

Chêneau braces differ conceptually from the Boston brace. While the Boston brace uses strategically placed pads in an effort to correct or halt progression or to induce some correction, Chêneau braces attempt spinal correction in three planes *via* an asymmetric design (Fig. **9**) in an attempt to achieve some curve reduction at weaning. To accomplish correction, each brace is created and applied according to an individual’s curve pattern [38].

**Fig. (10):**
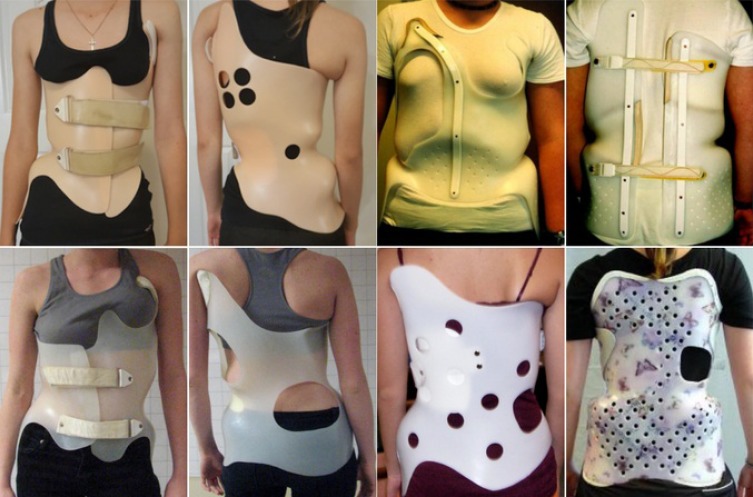
Examples of Chêneau braces produced in the U.S., Canada and Europe demonstrating that Chêneau braces may differ in size and appearance. [Courtesy of Dr. HR Weiss and Scoliosis 3DC, USA].

Like the scoliosis-specific exercise variants of Schroth, there are also versions of Chêneau bracing. The trademark voids at the concavities are perhaps one of the most identifiable aspects of a Chêneau brace [38]. In concept, each Chêneau brace will have numerous pressure points or zones to enhance correction in the frontal, sagittal and transverse planes and facilitate corrective rotational breathing. However, differences in correction effect depend on brace design, fitting practitioner, manufacturing technique, and the individual patient (Fig. ** 10**).

Weiss’s involvement in brace development can be traced to his tenure as medical director at the Katharina Schroth Clinic. Weiss recognized the necessity to make scoliosis bracing more patient-friendly and set out to develop braces that strive for the best correction effect. His initial attempts at improving bracing proved successful, and he went on to introduce CAD/CAM bracing using a system based on plaster models created by Rigo. Weiss tested the CAD versions of those plaster models at the German clinic, and fitted many of the patients [4, 41]. That early system became known as the RSC (Rigo System Chêneau) brace. To that point, the two men had collaborated to advance scoliosis bracing but since then have terminated their cooperative efforts.

**Fig. (11):**
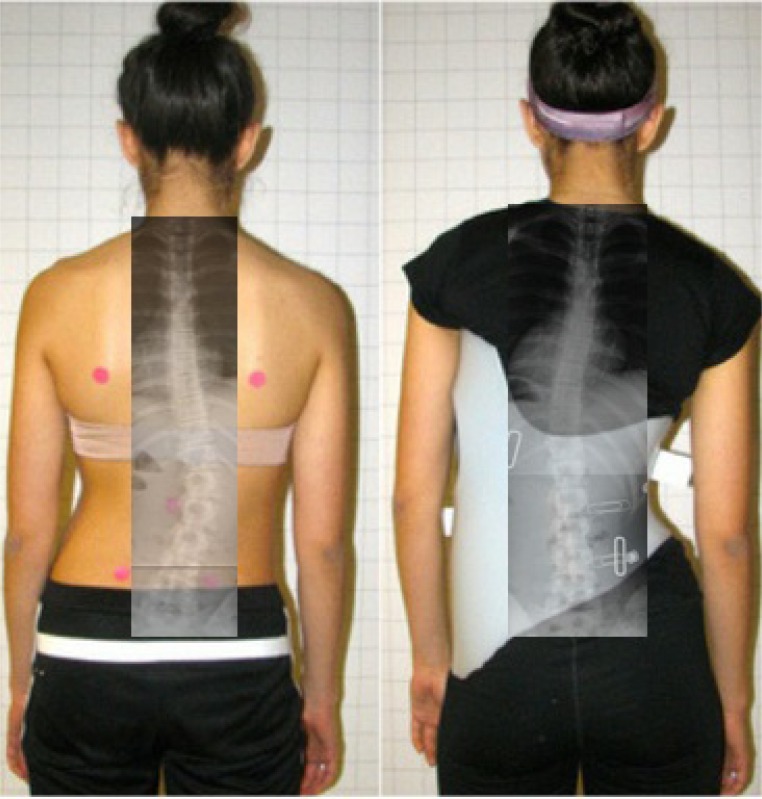
Overcorrection of a scoliosis curve in the Chêneau-Gensingen Brace® (GBW) 2015. [Courtesy of Scoliosis 3DC, USA].

**Fig. (12):**
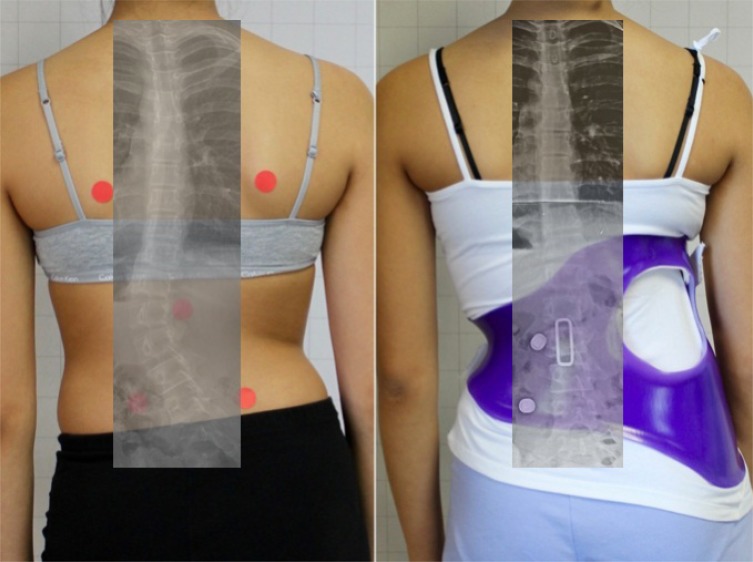
Complete correction of a 41º thoracolumbar scoliosis in the Chêneau-Gensingen Brace® in a 13-year-old girl, 2017. [Courtesy of Scoliosis 3DC, USA].

Weiss went on to develop and use the Chêneau-light™ brace until early 2011 [42]. His subsequent Chêneau bracing advancement, the Gensingen Brace® or Gensingen Brace by Weiss®/GBW), is currently designed and manufactured exclusively *via* CAD/CAM [43, 44]. The Gensingen Brace® attempts overcorrection whenever possible. Weiss also reduced the length of the brace and created the hemi-pelvis to make the Gensingen Brace® a smaller, more lightweight, wearer-friendly brace (Figs. **11**-**12**). It is designed to be a complementary supportive orthosis for use with all Schroth exercise protocols, or independently.

Weiss recognized vulnerabilities in quality control that inspired his efforts to improve bracing. He knew that bracing effectiveness varies significantly by manufacturer and orthotist. As a result, he developed his library of Gensingen braces based on the augmented Lehnert-Schroth classifications–the foundation of the original German Schroth method, which is still in use today [14]. Each Gensingen brace is designed to optimize the sagittal correction [44] and focus on inducing curve overcorrection whenever possible. The goal for each braced patient is Cobb angle reduction and improvement of spinal balance and postural appearance.

Today, thousands of Gensingen braces are created annually for a network of professionals who supply and fit the brace worldwide. Each brace is “standardized” which indicates that each brace is based on specific curve-pattern classifications (and subject to strict design parameters). Specifically, the CBA (Classification Based Approach) has seven basic curve patterns that were established as a sub-classification to the original Lehnert-Schroth classification (3- and 4-curve). Two additional patterns are used to address double thoracic curvatures and a thoracolumbar pattern with a structural high thoracic counter curve [44]. Weiss continually updates and improves his CAD/CAM system based on his professional experiences and on-going feedback from his network of bracing experts.

Other “Chêneau-style” bracing systems exist. Most of these have been created by individual orthotists who have developed their own versions of Chêneau braces, and others are presented to patients as modified according to the Chêneau principles. Many of these braces have unknown and unpublished results. This makes comparison of the different types of Chêneau braces virtually impossible and serves to underscore the benefits of standardization.

In-brace correction effect is essential to a successful result at the conclusion of brace treatment. Landauer *et al.* found that an initial in-brace correction of 40% or more results in a Cobb angle improvement of 7° or more at the end of brace treatment for compliant patients [45]. Regardless of the brace used, factors influencing potential outcomes are spinal flexibility, curve severity at the initiation of bracing, Risser stage, and wearing compliance.

## 4 RECENT HISTORY OF KYPHOSIS TREATMENT

Kyphosis can also be addressed by Schroth rehabilitation and bracing [10]. The original Schroth program included pelvic corrections and exercises to attempt to flatten the thoracic kyphosis for patients, even with a simultaneous presentation of scoliosis of 60º or more [5, 10].

**Fig. (13):**
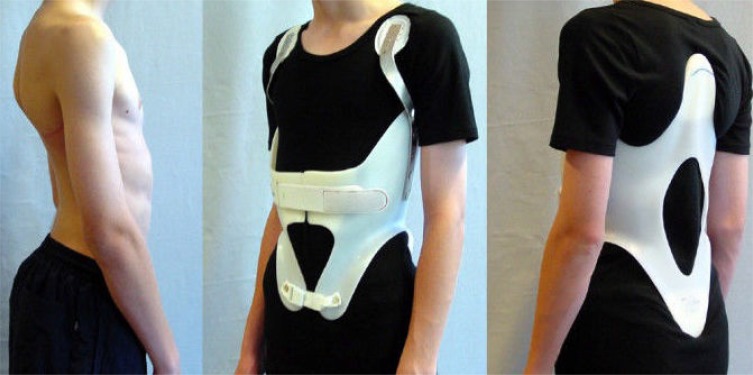
Kyphologic™ brace [49].

The Milwaukee brace has been shown to be effective in the treatment of a thoracic kyphosis [46]. In Europe, the Gschwend brace has been used successfully as well [47]; however, in recent years, Weiss introduced an updated brace design based on the correction principles of Katharina Schroth [10] known as the Kyphologic™ brace (Fig. **13**) [48, 49]. The Physio-logic™ brace, another brace developed for sagittal re-alignment, addresses thoracolumbar kyphosis and has been effective in reducing low back pain [50, 51].

For both rehabilitation and/or bracing, patient-centered care can lead to empowerment and improved compliance. For adolescents, time-sensitive treatment is imperative for spinal stabilization and potential improvement. While bracing is recommended until skeletal maturity, successful scoliosis rehabilitation management skills can be applied over a lifetime.
